# Cost-effectiveness of hydroxychloroquine *versus* placebo for hand osteoarthritis: economic evaluation of the HERO trial

**DOI:** 10.12688/f1000research.55296.1

**Published:** 2021-08-17

**Authors:** Sarah J Ronaldson, Ada Keding, Puvan Tharmanathan, Catherine Arundel, Sarah R Kingsbury, Philip G Conaghan, David J Torgerson

**Affiliations:** 1 York Trials Unit, Department of Health Sciences, University of York, York, YO10 5DD, UK; 2 Leeds Institute of Rheumatic and Musculoskeletal Medicine, and NIHR Leeds Biomedical Research Centre, University of Leeds, Leeds, LS7 4SA, UK

**Keywords:** economic evaluation, hand osteoarthritis, hydroxychloroquine, randomised controlled trial, cost-effectiveness analysis, cost-utility analysis

## Abstract

**Background:** An economic evaluation alongside the Hydroxychloroquine Effectiveness in Reducing symptoms of hand Osteoarthritis (HERO) trial was undertaken to assess the cost-effectiveness of hydroxychloroquine compared with placebo for symptomatic treatment of hand osteoarthritis for patients with at least moderate hand pain and inadequate response to current therapies.

**Methods:** A trial-based cost–utility analysis was undertaken from the perspective of the UK National Health Service and Personal Social Services over a 12-month time horizon, using evidence from 248 participants included in the HERO trial, conducted in England. Patient-level data were collected prospectively over a 12-month period, using participant-completed questionnaires and investigator forms, to collect healthcare utilisation, costs and quality-adjusted life years (QALYs) using the EQ-5D-5L. The base-case analysis was conducted on an intention-to-treat basis and used multiple imputation methods to deal with missing data. Results were presented in terms of incremental cost-effectiveness ratios (incremental cost per QALY) and net health benefit, with uncertainty surrounding the findings explored using cost-effectiveness acceptability curves.

**Results:** The base-case analysis estimated slightly lower costs on average (−£11.80; 95% confidence interval (CI) −£15.60 to −£8.00) and marginally fewer QALYs (−0.0052; 95% CI −0.0057 to −0.0047) for participants in the hydroxychloroquine group versus placebo group at 12 months. The resulting incremental cost-effectiveness ratio of £2,267 per QALY lost indicated that although costs were saved, health-related quality of life was lost. Even assuming symmetrical preferences regarding losses and gains for health benefits, the findings do not fall within the cost-effective region. Similar findings arose for analyses conducted from the societal perspective and using complete cases only.

**Conclusions:** This economic evaluation indicates that hydroxychloroquine is unlikely to provide a cost-effective pain relief option for improving health-related quality of life in adult patients with moderate-to-severe hand osteoarthritis.

## Introduction

Hand osteoarthritis (OA) is a common, chronic disease, with newly diagnosed cases of hand OA estimated to occur in 2.4 per 1000 at-risk adults aged 45 and over in England each year. ^1^ More generally, OA poses an increasing burden to health services, ^2^ and is associated with substantial costs. ^3– 5^ These high costs arise as direct health-related costs, in the form of long-term pain control treatments, surgery and rehabilitation, and also indirect costs, such as productivity loss and costs relating to home care or childcare. ^4, 5^ The annual cost in the UK for topical nonsteroidal anti-inflammatories (NSAIDs) and oral NSAIDs, two of the most commonly used pharmacological therapies for OA, was estimated to be £19.2 million and £25.7 million respectively, in 2010 prices. ^3^ In addition to the financial burden, OA also poses a considerable burden in terms of morbidity, sometimes considered as ‘intangible costs’, through a reduction in health-related quality of life experienced by OA patients. ^3^

In order to improve the quality of life of hand OA patients, different management options are available; however, there is a paucity of effective treatments, with side effects often accompanying treatment. ^6– 8^ An option that has been explored in the past leading to unlicensed use is hydroxychloroquine (HCQ), which is established as a treatment for rheumatoid arthritis with acceptable safety. ^9, 10^ The Hydroxychloroquine Effectiveness in Reducing symptoms of hand Osteoarthritis (HERO) trial assessed the use of HCQ *versus* placebo as a treatment for people with at least moderate symptomatic and radiographic hand OA and inadequate response to current therapies (including NSAIDs and opioids). The clinical findings of the HERO trial demonstrated that HCQ was no more effective than placebo for pain relief when added to usual care, shown using a primary outcome of hand pain severity over the past two weeks as measured on an eleven-point (0 to 10) numerical rating scale (NRS) at six months. ^11^ Despite the lack of clinical effectiveness, it is useful to summarise the economic findings to provide evidence that can help guide the efficient allocation of healthcare resources, and also to present the healthcare resource use and health-related quality of life associated with the population of patients included in the trial. Such findings may be useful for future evaluations.

The aim of this economic analysis was to evaluate the cost-effectiveness of HCQ *versus* placebo as a symptomatic treatment for patients over the age of 18 with at least moderately symptomatic hand OA and inadequate response to current therapies. The economic analysis was conducted over a 12-month time horizon from the perspective of the UK National Health Service (NHS) and Personal Social Services (PSS), and comprised (i) a cost-utility analysis, in terms of the cost per quality-adjusted life year (QALY) and (ii) a cost-effectiveness analysis, in terms of the cost per unit of reduction in pain score.

## Methods

### Overview

The HERO trial was a randomised, double-blind, placebo-controlled trial with an economic evaluation conducted alongside. Patients with symptomatic and radiographic hand OA aged 18 years or over were recruited from 13 primary and secondary care centres in England; description of the study design and interventions are available in full elsewhere. ^11, 12^ A total of 248 participants were included in the trial; participants were aged 62.7 years on average (ranging from 40 to 88 years), with 81.9% being female. The trial involved 12-month follow up of the use of HCQ (200–400 mg) or placebo, in addition to ongoing usual care, where participants were randomised on a 1:1 basis. The study was registered with International Standard Randomised Controlled Trial Number ISRCTN91859104 on 17 October 2012, and received approval by Leeds East Research Ethics Committee (12/YH/0151) and the UK Medicines and Health Regulatory Authority. All participants provided written informed consent prior to screening and involvement in the trial.

A within-trial economic analysis was undertaken from the perspective of the UK NHS and PSS, with results presented in terms of the incremental cost per QALY (cost–utility analysis), and the cost per unit of reduction in pain score (cost-effectiveness analysis), which used the trial’s primary outcome of hand pain severity. Patient-level data were collected for costs (healthcare resource use, medication use, and of HCQ drug) and health outcomes (EuroQol EQ-5D-5L and hand pain severity) over the 12-month follow up period, using questionnaires completed by participants at baseline, six months and 12 months. The base-case analysis was undertaken using multiple imputation to deal with missing data, with complete case analysis explored as a sensitivity analysis. A secondary analysis from the broader societal perspective was also conducted.

### Health outcomes

QALYs were estimated by participants’ completion of the EQ-5D-5L (EuroQol Group, Rotterdam, The Netherlands) ^13, 14^ in self-completed questionnaires at baseline, six and 12 months follow up. The EQ-5D-5L is a preference-based measure that provides a descriptive profile of an individual’s health state ^15^ and comprises five dimensions (mobility, self-care, usual activities, pain/discomfort and anxiety/depression) which each have five possible levels of response (no, slight, moderate, or severe problems, or unable to/extreme problems). The crosswalk value set developed by van Hout *et al.* ^16^ was utilised for the estimation of utilities. A utility score of one indicates perfect health, a utility of zero indicates states equivalent to death, and negative scores indicate states considered to be worse than death. QALYs were calculated by plotting the utility scores at each of the three time points and estimating the area under the curve, ^17, 18^ with adjustment made for baseline utility. ^19^ In addition to the evaluation of QALYs *via* a cost-utility analysis, the primary clinical outcome of the trial, hand pain severity, was also investigated via a cost-effectiveness analysis. Specifically, the reduction in hand pain severity was measured using a NRS (0 to 11, where higher scores represent worse levels of pain) and assessed by calculating the difference in hand pain NRS at 6 months and hand pain NRS at baseline.

### Resource use and unit costs

Participants were asked about their utilisation of healthcare services in relation to their hand or hand pain via self-completed questionnaires at baseline, six and 12 months. Resource use items included in the questionnaire aimed to represent relevant services used by patients with hand OA, specifically within primary care (visits to the general practitioner (GP), nurse, and other primary care services), community care (physiotherapist and occupational therapist visits, and other community care services) and in the hospital setting (outpatient attendances, day case visits, and accident and emergency attendances). Total resource use per participant for the duration of the 12-month trial period was calculated by multiplying each resource use item by the corresponding unit cost ( Table 1); unit costs were derived from established national costing sources. ^20, 21^ Participants were also asked *via* the study questionnaires about personal expenses, namely travel and childcare costs relating to healthcare appointments and over-the-counter medication costs, which fed into the secondary analysis, undertaken from a broader societal perspective. Unit costs were not required for these costs, as participants were asked to specifically state the amount of money spent on these, rather than the number of resources used.

**Table 1.** Unit costs of healthcare resource use.

Item	Unit of measurement	Unit cost	Source
Hospital outpatient clinic visit	Per clinic visit ^a^	£114.50	( 20)
Accident & Emergency visit	Per attendance	£140.59	( 20)
Other outpatient visit ^b^	Per visit	£115.88	( 20)
GP visit at GP practice	Per patient contact (surgery) lasting 11.7 mins	£44.00	( 21)
GP visit at home	Per home visit (11.4 mins) plus 12 mins travel time	£88.92	( 21)
Nurse visit at GP practice	Per 15.5 min appointment (based on £43 per hour)	£11.11	( 21)
Other primary care visit ^c^	Per visit	£33.30	( 20, 21)
Physiotherapist visit	Per hour	£38.00	( 21)
Occupational therapist visit	Per hour	£44.00	( 21)
Other community care visit ^d^	Per visit	£66.57	( 20)

GP general practitioner. ^a^ Based on average of total outpatient attendances; ^b^ Based on average of most commonly reported reasons for other outpatient visit (i.e. physiotherapy, trauma & orthopaedics, general surgery, MRI, x-ray); ^c^ Based on average of most commonly reported reasons for other primary care visit (i.e. blood tests, counselling, nurse visit); ^d^ Based on average of most commonly reported reasons for other community care visit (i.e. acupuncture, physiotherapist, podiatrist); where costs were not available, costs were assumed to be the same as similar types of visit.

### Medications

Participants received a daily dose of HCQ in either 200, 300 or 400 mg (for 300 mg, alternating doses of 200 mg and 400 mg were taken); for costing purposes, participants were assumed to continue on the same dose throughout the study period (which was, in fact the case, with the exception of one participant). Unit costs of HCQ and medications used by participants, as recorded by study investigators, were obtained from the British National Formulary. ^22^ The HCQ costing was based on the net price of HCQ sulfate 200 mg (60-tablet pack) being £5.15. ^23^ The cost of HCQ use over the 12-month period was applied, unless there was information recorded regarding the participant stopping/withdrawing from treatment. The cost of placebo tablets represented a research cost and hence excluded from the costings. Information regarding participants’ use of oral and topical medication was recorded by study investigators at regular intervals during the trial period; investigator forms at 3, 6, 9 and 12 months were used for the medication costing. To attach costs to participants’ medication use, each medication was categorised ( *e.g.* oral opioid, topical NSAID, antidepressant/neuropathic therapy), and within each category the average cost of the most commonly occurring medications was applied. A total medication cost per participant was generated by summing the cost of all medications used by the participant.

### Data analysis

The base-case analysis was conducted on an intention-to-treat (ITT) basis; the two groups (HCQ *versus* placebo) were compared according to their initial random allocation, regardless of whether protocol deviations or withdrawals took place. Cost and outcome data were collected prospectively during the study and compared for the two groups over 12 months, hence discounting was not required. Costs are presented in UK £ sterling at 2015 prices and the analysis was undertaken in Stata 13© (StataCorp 2013, TX, USA) (RRID:SCR_012763); an open-access alternative is R (RRID:SCR_001905). The base-case analysis utilised a dataset generated *via* multiple imputation with chained equations and predictive mean matching to deal with missing data, based on the assumption that data were missing at random. The imputation model included baseline hand pain severity, concomitant analgesic use, average grip strength, body mass index (BMI), age and gender (all consistent with the clinical primary analysis model), baseline costs and baseline utility. A complete case analysis was also undertaken as a sensitivity analysis, where patients with any missing data were excluded, and available case analysis was used for initial exploration of the data.

Mean differences in costs and QALYs were compared for the two groups to assess the cost-effectiveness of HCQ *versus* placebo; estimates were produced using seemingly unrelated regression equations, with 95% confidence intervals estimated using bias corrected and accelerated bootstrap methods. The regression model adjusted for baseline utility and covariates consistent with those used in the trial’s statistical analysis. Differences between the groups were found to be statistically significant if P<0.05 and are presented alongside confidence intervals around the differences in costs and outcomes. Incremental cost-effectiveness ratios (ICERs) were used to present the findings, where appropriate, thereby indicating the additional cost per extra unit of benefit, calculated by dividing the mean difference in costs between trial groups by the mean difference in health outcomes. An ICER is compared with cost-effectiveness threshold values, to determine whether the intervention represents value for money. The NICE cost-effectiveness threshold for the UK ranges between £20,000 and £30,000 per QALY, ^24^ hence both of these thresholds featured in the analysis. Results are also presented in terms of net health benefit (NHB) ^25^ to aid interpretation of the findings; a positive NHB indicates an intervention is cost-effective, based on the cost-effectiveness threshold under consideration. The decision uncertainty surrounding the cost-effectiveness findings was explored using cost-effectiveness planes and cost-effectiveness acceptability curves (CEACs). ^26^ A total of 5,000 estimates of incremental costs and incremental effects were generated, using non-parametric bootstrapping, and plotted on the cost-effectiveness plane. CEACs have been used to depict the probability of HCQ being cost-effective relative to placebo for a range of cost-effectiveness thresholds.

In addition to the cost-utility analysis described above, the cost-effectiveness analysis that explored the cost per unit of reduction in hand pain score utilised a mixed model, in line with the statistical model used for the clinical effectiveness analysis. This model accounted for baseline covariates and used an exchangeable covariance structure to account for the correlation of observations from the same patients over time.

## Results

A total of 248 patients were recruited to the trial, of whom, 124 were randomised to receive HCQ and 124 to placebo. Complete utility data were available for 183 (73.8%) participants: 88 (71.0%) HCQ participants and 95 (76.6%) placebo participants, that is, all five dimensions of the EQ-5D-5L were completed at the three timepoints. Complete economic data, *i.e.* for both utilities and costs, were available for 76 participants (30.7%; 42 (33.9%) HCQ participants and 34 (27.4%) placebo participants). No deaths were reported during the trial period.

### Resource use and costs

Mean healthcare resource use is summarised in Table 2 by group and timepoint, for all available cases. Most notable health services used by participants over the duration of the trial (sum of 6 month and 12 month resource use) were for: 2.7 GP visits (at GP practice), 2.2 outpatient clinic visits, 1.1 nurse visits (at GP practice), and 0.7 physiotherapist visits per participant, on average. At 12 months, participants in the HCQ group had fewer hospital outpatient visits, GP visits and nurse visits, but higher physiotherapist, occupational therapist, and accident and emergency (A&E) visits. In terms of the total mean cost for each resource use item, Table 3 summarises this according to group and the mean difference between groups, based on all available cases. Costs were found to be lower for the HCQ group, on average, for visits to the GP (at GP practice), nurse, A&E and hospital outpatient attendances, but were higher for GP visits (at home), physiotherapist and occupational therapist visits. However, none of the differences between groups were statistically significant.

**Table 2.** Mean healthcare resource use, based on all available cases.

Type of resource use	HCQ (n = 124)			Placebo (n = 124)		
	Mean (SD)	Missing (%)		Mean (SD)	Missing (%)	
**Hospital outpatient visit**						
Baseline	2.05 (2.57)	1	0.8%	1.51 (2.14)	3	2.4%
6 months	1.07 (1.58)	17	13.7%	1.08 (1.61)	22	17.7%
12 months	0.97 (1.53)	33	26.6%	1.29 (2.41)	29	23.4%
**Accident & Emergency visit**						
Baseline	0.13 (0.72)	1	0.8%	0.06 (0.35)	1	0.8%
6 months	0.02 (0.14)	18	14.5%	0.09 (0.32)	23	18.5%
12 months	0.05 (0.23)	32	25.8%	0.03 (0.17)	28	22.6%
**Other outpatient visit 1**						
Baseline	0.68 (1.80)	13	10.5%	0.71 (2.61)	12	9.7%
6 months	0.29 (0.83)	24	19.4%	0.33 (1.71)	30	24.2%
12 months	0.60 (2.39)	39	31.5%	0.40 (1.04)	39	31.5%
**Other outpatient visit 2**						
Baseline	0.27 (0.85)	50	40.3%	0.11 (0.39)	48	38.7%
6 months	0.09 (0.39)	43	34.7%	0.13 (0.62)	49	39.5%
12 months	0.19 (0.58)	57	46.0%	0.25 (0.81)	68	54.8%
**GP visit at GP practice**						
Baseline	2.46 (2.77)	0	0.0%	1.95 (3.98)	4	3.2%
6 months	1.50 (1.90)	17	13.7%	1.28 (1.76)	21	16.9%
12 months	1.14 (1.33)	32	25.8%	1.67 (1.88)	29	23.4%
**GP visit at home**						
Baseline	0.01 (0.09)	1	0.8%	0.02 (0.13)	1	0.8%
6 months	0.02 (0.19)	18	14.5%	0.01 (0.10)	21	16.9%
12 months	0.01 (0.10)	32	25.8%	0.02 (0.14)	29	23.4%
**Nurse visit at GP practice**						
Baseline	0.80 (1.49)	1	0.8%	0.42 (0.76)	3	2.4%
6 months	0.67 (2.00)	18	14.5%	0.48 (1.10)	23	18.5%
12 months	0.48 (0.99)	34	27.4%	0.64 (1.02)	30	24.2%
**Other primary care visit 1**						
Baseline	0.19 (0.76)	9	7.3%	0.15 (0.75)	8	6.5%
6 months	0.25 (1.30)	20	16.1%	0.09 (0.41)	27	21.8%
12 months	0.09 (0.42)	38	30.6%	0.07 (0.39)	30	24.2%
**Other primary care visit 2**						
Baseline	0.10 (0.58)	35	28.2%	0.01 (0.11)	41	33.1%
6 months	0.01 (0.11)	41	33.1%	0.01 (0.11)	45	36.3%
12 months	0 (0)	58	46.8%	0 (0)	57	46.0%
**Physiotherapist visit**						
Baseline	0.75 (1.96)	1	0.8%	0.69 (2.05)	2	1.6%
6 months	0.22 (0.87)	19	15.3%	0.30 (1.16)	22	17.7%
12 months	0.48 (2.25)	33	26.6%	0.32 (1.36)	28	22.6%
**Occupational therapist visit**						
Baseline	0.35 (1.11)	2	1.6%	0.12 (0.60)	5	4.0%
6 months	0.04 (0.19)	20	16.1%	0.08 (0.52)	22	17.7%
12 months	0.16 (0.60)	34	27.4%	0.03 (0.18)	30	24.2%
**Other community care visit 1**						
Baseline	0.03 (0.16)	4	3.2%	0.40 (3.05)	5	4.0%
6 months	0.11 (0.71)	20	16.1%	0.08 (0.58)	24	19.4%
12 months	0.02 (0.21)	36	29.0%	0.05 (0.37)	28	22.6%
**Other community care visit 2**						
Baseline	0 (0)	34	27.4%	0 (0)	36	29.0%
6 months	0.05 (0.34)	37	29.8%	0 (0)	40	32.3%
12 months	0 (0)	57	46.0%	0 (0)	52	41.9%

HCQ hydroxychloroquine; SD standard deviation; GP general practitioner.

**Table 3.** Total mean costs based on all available cases, up to 12 month follow up

Cost item	Total mean cost £ (SD)		Mean difference (HCQ - Placebo) (95% CI)
	HCQ	Placebo	
**Hospital outpatient visit**	226.48 (273.62) N = 91	269.63 (406.10) N = 93	−43.15 (−144.09, 57.80)
**Accident & Emergency visit**	9.27 (35.08) N = 91	16.45 (61.36) N = 94	−7.18 (−21.74, 7.38)
**Other outpatient visit 1**	100.15 (300.38) N = 81	88.79 (266.45) N = 77	11.35 (−78.05, 100.75)
**Other outpatient visit 2**	36.48 (83.70) N = 54	45.81 (126.79) N = 43	−9.33 (−51.92, 33.25)
**GP visit at GP practice**	105.70 (99.58) N = 92	131.06 (136.57) N = 94	−25.37 (−60.01, 9.27)
**GP visit at home**	2.93 (20.75) N = 91	2.84 (15.71) N = 94	0.09 (−5.23, 5.42)
**Nurse visit at GP practice**	11.23 (20.81) N = 89	12.70 (20.99) N = 91	−1.46 (−7.61, 4.69)
**Other primary care visit 1**	11.50 (50.25) N = 84	5.74 (22.83) N = 87	5.76 (−5.96, 17.47)
**Other primary care visit 2**	0.58 (4.41) N = 57	0.64 (4.62) N = 52	−0.06 (−1.77, 1.66)
**Physiotherapist visit**	27.33 (98.73) N = 89	23.85 (68.02) N = 94	3.47 (−21.14, 28.09)
**Occupational therapist visit**	9.10 (30.85) N = 87	4.78 (24.80) N = 92	4.32 (−3.91, 12.56)
**Other community care visit 1**	10.18 (53.87) N = 85	5.79 (32.21) N = 92	4.39 (−8.66, 17.44)
**Other community care visit 2**	4.75 (27.98) N = 56	0 (0) N = 59	4.75 (−2.46, 11.97)
**Medications**	282.16 (234.71) N = 124	300.17 (232.36) N = 124	−18.01 (−76.43, 40.41)
**Secondary analysis costs:**			
Over-the-counter drugs	25.15 (46.27) N = 87	23.44 (39.81) N = 92	1.71 (−11.01, 14.42)
Child care	6.17 (58.50) N = 90	0 (0) N = 92	6.17 (−5.87, 18.20)
Travel to appointments	18.42 (67.25) N = 88	11.13 (20.14) N = 91	7.29 (−7.25, 21.83)

HCQ hydroxychloroquine; SD standard deviation; CI confidence interval; GP general practitioner.

The mean (standard deviation, SD) cost of HCQ for the 12-month period was £42.14 (£16.23), with HCQ costs estimated based on the following: seven participants prescribed a daily dose of 200 mg, 85 receiving 300 mg and 32 receiving 400 mg HCQ daily. A total of 90 HCQ participants received HCQ for the 12-month trial duration, whilst 34 received HCQ for durations less than this (ranging from 15 days to 308 days). Mean (SD) medication costs over the 12-month period were £282 (£235) for HCQ participants and £300 (£232) for placebo participants, for all available cases over the 12-month follow up. Hence, larger components of the total cost derived from hospital outpatient clinic appointments, GP visits (at GP practice) and medication costs, with costs of the drug, occupational therapist and physiotherapist visits being smaller cost drivers.

### Health outcomes

*Utility and quality-adjusted life years*

Estimation of the proportion of participants who reported the EQ-5D-5L levels (1 to 5) by dimension and group identified that the majority of participants (at least 95%) reported having problems in terms of pain/discomfort at all time points in both groups, whereas problems with self-care were reported for approximately a quarter to a third of participants ( Table 4). Baseline utility was slightly higher in the HCQ group (0.615 *versus* 0.612 for HCQ and placebo, respectively) and the differences found between the groups at both 6 and 12 months were found to be very small ( Table 5). The difference in QALYs at 12 months (HCQ−placebo), controlling for baseline utility, was 0.0012 (95% CI: −0.0251 to 0.0276), for available cases (n = 88 HCQ, n = 95 placebo). EQ-5D visual analogue scale (VAS) scores were found to be similar at baseline on average (72.4 for HCQ; 73.9 for placebo), and by 6 months, there was an increase for the HCQ group (to 74.5) compared with a slight reduction for the placebo group (73.6). However, by 12 months, the VAS score increased for both groups: 74.1 and 75.1 for the HCQ and placebo groups, respectively.

**Table 4.** Proportion reporting EQ-5D-5L levels 1 to 5 by dimension, group and time point.

EQ-5D dimension	Health state severity ^**a**^	Baseline				6 months				12 months			
		HCQ		Placebo		HCQ		Placebo		HCQ		Placebo	
**Mobility**	**Level 1**	52	41.9%	52	41.9%	45	36.3%	37	29.8%	37	29.8%	40	32.3%
	**Level 2**	37	29.8%	27	21.8%	27	21.8%	31	25.0%	26	21.0%	24	19.4%
	**Level 3**	27	21.8%	31	25.0%	26	21.0%	25	20.2%	22	17.7%	23	18.5%
	**Level 4**	8	6.5%	12	9.7%	8	6.5%	10	8.1%	7	5.6%	9	7.3%
	**Level 5**	0	0.0%	0	0.0%	0	0.0%	0	0.0%	0	0.0%	1	0.8%
	**Missing**	0	0.0%	2	1.6%	18	14.5%	21	16.9%	32	25.8%	27	21.8%
**No. reporting any problems**		72		70		61		66		55		57	
		58.06%		57.38%		57.55%		64.08%		59.78%		58.76%	
**Self-care**	**Level 1**	87	70.2%	77	62.1%	79	63.7%	73	58.9%	70	56.5%	73	58.9%
	**Level 2**	24	19.4%	30	24.2%	16	12.9%	17	13.7%	13	10.5%	15	12.1%
	**Level 3**	12	9.7%	13	10.5%	11	8.9%	9	7.3%	8	6.5%	8	6.5%
	**Level 4**	1	0.8%	2	1.6%	0	0.0%	4	3.2%	1	0.8%	2	1.6%
	**Level 5**	0	0.0%	0	0.0%	0	0.0%	0	0.0%	0	0.0%	0	0.0%
	**Missing**	0	0.0%	2	1.6%	18	14.5%	21	16.9%	32	25.8%	26	21.0%
**No. reporting any problems**		37		45		27		30		22		25	
		29.84%		36.89%		25.47%		29.13%		23.91%		25.51%	
**Usual activities**	**Level 1**	27	21.8%	25	20.2%	38	30.6%	31	25.0%	33	26.6%	32	25.8%
	**Level 2**	50	40.3%	55	44.4%	33	26.6%	36	29.0%	35	28.2%	33	26.6%
	**Level 3**	37	29.8%	34	27.4%	29	23.4%	28	22.6%	19	15.3%	27	21.8%
	**Level 4**	10	8.1%	7	5.6%	7	5.6%	8	6.5%	4	3.2%	6	4.8%
	**Level 5**	0	0.0%	1	0.8%	0	0.0%	0	0.0%	1	0.8%	0	0.0%
	**Missing**	0	0.0%	2	1.6%	17	13.7%	21	16.9%	32	25.8%	26	21.0%
**No. reporting any problems**		97		97		69		72		59		66	
		78.23%		79.51%		64.49%		69.90%		64.13%		67.35%	
**Pain/discomfort**	**Level 1**	1	0.8%	2	1.6%	0	0.0%	0	0.0%	1	0.8%	5	4.0%
	**Level 2**	14	11.3%	14	11.3%	33	26.6%	29	23.4%	28	22.6%	37	29.8%
	**Level 3**	78	62.9%	75	60.5%	53	42.7%	54	43.5%	43	34.7%	33	26.6%
	**Level 4**	30	24.2%	28	22.6%	18	14.5%	20	16.1%	17	13.7%	22	17.7%
	**Level 5**	1	0.8%	2	1.6%	3	2.4%	0	0.0%	2	1.6%	1	0.8%
	**Missing**	0	0.0%	3	2.4%	17	13.7%	21	16.9%	33	26.6%	26	21.0%
**No. reporting any problems**		123		119		107		103		90		93	
		99.19%		98.35%		100.00%		100.00%		98.90%		94.90%	
**Anxiety/depression**	**Level 1**	64	51.6%	73	58.9%	64	51.6%	67	54.0%	53	42.7%	62	50.0%
	**Level 2**	35	28.2%	30	24.2%	22	17.7%	28	22.6%	28	22.6%	26	21.0%
	**Level 3**	21	16.9%	19	15.3%	16	12.9%	8	6.5%	7	5.6%	9	7.3%
	**Level 4**	2	1.6%	0	0.0%	4	3.2%	0	0.0%	3	2.4%	1	0.8%
	**Level 5**	1	0.8%	0	0.0%	0	0.0%	0	0.0%	0	0.0%	0	0.0%
	**Missing**	1	0.8%	2	1.6%	18	14.5%	21	16.9%	33	26.6%	26	21.0%
**No. reporting any problems**		59		49		42		36		38		36	
		47.97%		40.16%		39.62%		34.95%		41.76%		36.73%	

HCQ hydroxychloroquine; ^a^ level 1 - no problems; level 2 – slight problems; level 3 – moderate problems; level 4 – severe problems; level 5 – extreme problems.

**Table 5.** Summary of EQ-5D-5L utility scores at each time point (all available cases).

Utility	HCQ (n = 124)		Placebo (n = 124)		Unadjusted mean difference (HCQ - Placebo) (95% CI)	Adjusted mean difference ^**a**^ (HCQ - Placebo) (95% CI)
Follow up	N	Mean (SD)	N	Mean (SD)		
**Baseline**	123	0.615 (0.176)	121	0.612 (0.180)	0.0026 (−0.042, 0.047)	
**6 months**	106	0.635 (0.198)	103	0.641 (0.171)	−0.0068 (−0.057, 0.044)	−0.0008 (−0.035, 0.033)
**12 months**	90	0.638 (0.206)	97	0.642 (0.204)	−0.0041 (−0.063, 0.055)	−0.0055 (−0.052, 0.411)

HCQ hydroxychloroquine; SD standard deviation; CI confidence interval.

^a^ The difference at 6 and 12 months is adjusted for baseline utility.

*Hand pain score*

The differences between treatment groups in terms of hand pain score were found to be small at each follow up time point and not statistically significant. At the primary endpoint of 6 months, patients in the HCQ group scored on average 0.16 points higher on the pain scale than those in the placebo group, therefore indicating worse pain for HCQ than placebo patients. As there was not a reduction found in terms of pain, it was not considered meaningful to calculate the ICER as originally intended, as it would have instead represented the cost per additional unit of pain.

### Cost-utility analysis and uncertainty

The use of HCQ *versus* placebo was associated with a cost saving of £11.80 per participant, on average, in the base-case analysis ( Table 6). In terms of the effect of HCQ on health-related quality of life, only marginal differences in QALYs were demonstrated. The base-case analysis found 0.005 fewer QALYs for participants in the HCQ group compared to the placebo group, on average, over the 12-month time horizon. It was therefore appropriate to report the results in terms of the cost per QALY lost, rather than per QALY gained that is more commonly seen; the resulting ICER showed cost savings of £2,267 per QALY lost, which implies that £2,267 would need to be saved in order to justify a loss of one QALY, *i.e.* costs are being saved but health is being lost. The NHB was found to be negative: −£92.30 (95% CI: −£102.11 to −£82.49) and −£144.34 (95% CI: −£158.67 to −£130.02) for the £20,000 and £30,000 cost-effectiveness thresholds, respectively.

**Table 6.** Cost-utility analysis results: for base-case analysis, secondary analysis and complete case analysis

	Base-case ^**a**^ (MI)	Societal perspective (MI)	Complete case analysis
**Incremental mean cost (£)** ^**b**^	−11.80 (−15.60, −8.00)	−2.66 (−6.14, 0.81)	−50.95 (−540.42, 438.51)
**Incremental mean QALYs** ^**b**^	−0.0052 (−0.0057, −0.0047)	−0.0101 (−0.0105, −0.0096)	−0.0054 (−0.0675, 0.0567)
**ICER (£): cost per QALY**	2,267 ^**c**^	265 ^**c**^	9,417 ^**c**^
**NHB (£) (95% CI)** ^**b**^ **, based on £30,000/QALY**	−144.34 (−158.67, −130.02)	−299.16 (−313.16, −285.17)	−144.30 (−169.15, −119.45)
**NHB (£) (95% CI)** ^**b**^ **, based on £20,000/QALY**	−92.30 (−102.11, −82.49)	−198.56 (−208.08, −189.03)	−81.67 (−98.21, −65.14)

MI multiple imputation; QALYs quality-adjusted life years; ICER incremental cost-effectiveness ratio; NHB net health benefit.

^a^ Adjusted for all covariates, including baseline utility & cost.

^b^ Difference between groups (HCQ – placebo) and 95% bias corrected and accelerated confidence intervals were estimated using seemingly unrelated regression.

^c^ Cost per QALY lost, which implies that £2,267 would need to be saved in order to justify a loss of one QALY, i.e. costs are being saved but health is being lost.

**Figure 1.**
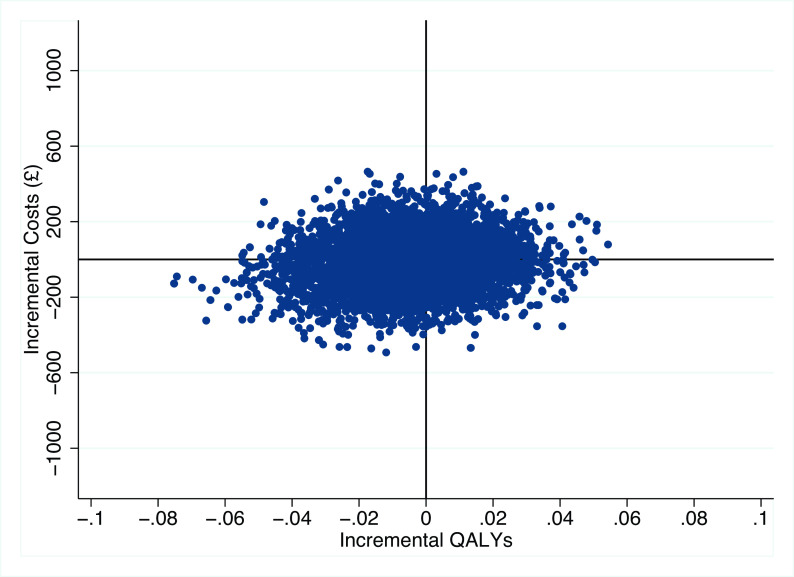
Scatter plot on the cost-effectiveness plane: incremental costs and incremental QALYs (base-case).

**Figure 2.**
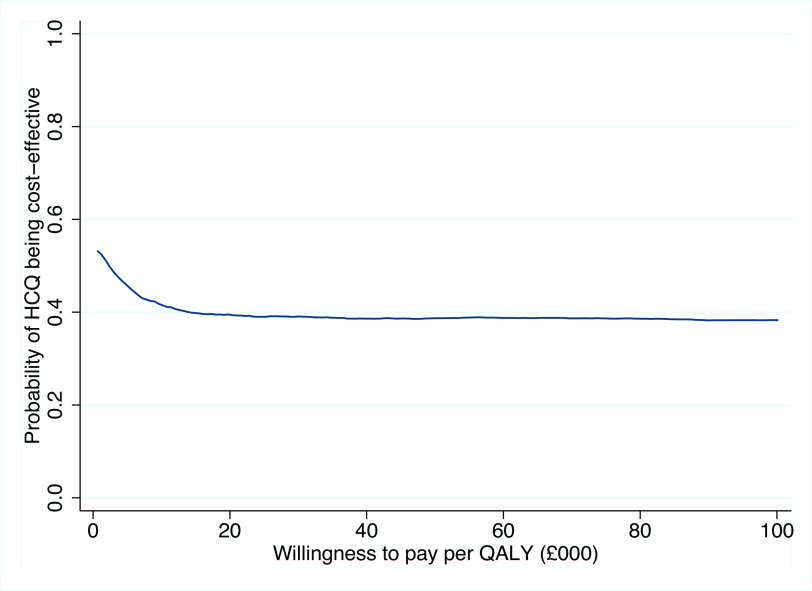
Cost-effectiveness acceptability curve (base-case).

Figure 1 illustrates the 5,000 bootstrap sample estimates, which are spread across the four quadrants of the cost-effectiveness plane quite evenly. The probability of HCQ being cost-effective for different willingness to pay thresholds is shown in Figure 2. For the NICE threshold of £20,000 per QALY, the probability of HCQ being cost-effective (under the base-case scenario) is 0.40, and similar at 0.39 for a threshold of £30,000 per QALY.

### Secondary analysis

The secondary analysis undertaken from a societal perspective (incorporating personal costs for over-the-counter medication, childcare and travel for appointments) showed participants in the HCQ group incurred marginally lower mean costs (−£2.66; 95% CI: −£6.14 to £0.81), and slightly fewer QALYs (−0.010; 95% CI: −0.011 to −0.010) compared with placebo. The marginal cost saving for the HCQ group was not statistically significant. The resulting ICER was £265 per QALY lost, with a negative NHB at both the £20,000 threshold (−£199) and the £30,000 threshold (−£299).

### Sensitivity analysis: complete case analysis

The findings of the cost-utility analysis remained in the same direction for the complete case analysis, which comprised 76 participants (31% of total participants) who had complete economic data profiles, although a larger cost difference was found than in the base-case. Participants in the HCQ group incurred costs that were £50.95 lower than those in the placebo group, and 0.005 fewer QALYs, *i.e.* similar to the QALY difference in the base-case. However, the cost and QALY differences were not found to be statistically significant for the complete case analysis.

## Discussion

This economic evaluation found that HCQ was associated with lower costs (mean cost reduction of −£11.80; 95% CI: −£15.60 to −£8.00), produced a marginally smaller mean QALY gain (−0.005 QALYs; 95% CI: −0.006 to −0.005 QALYs) and scored a worse level of pain on the hand pain scale at 6 months than placebo, on average. Hence, the differences in both costs and effectiveness were very small between the two groups. The corresponding ICER indicated a cost saving of £2,267 per QALY lost, implying that £2,267 would need to be saved in order to justify a loss of one QALY. Since the results showed a saving of £2,267 per QALY lost, HCQ is unlikely to be recommended from an economic perspective for hand OA management. The decision rule used in the UK is that where an intervention costs less than £20,000 per QALY when compared to its comparator, it is considered cost-effective. If we assume that decision makers have symmetrical preferences regarding losses and gains, ^27^ then (where findings are in terms of the cost per QALY lost) an intervention would be considered cost effective if it could achieve cost savings greater than £20,000 per QALY forgone, when compared with the alternative. Considering this on the cost-effectiveness plane, a larger ICER in the south-west quadrant of the plane indicates that a larger cost saving is associated with each unit of forgone health benefit, therefore an ICER that exceeds the cost-effectiveness threshold is preferred. ^28^

The study adds to the evidence base around management of hand OA, in finding that HCQ was not a cost-effective option and no more effective compared with placebo for pain relief of radiographic OA patients who have moderate-to-severe hand pain. Thereby, providing further evidence to reconsider continued use of HCQ in this patient group in line with the clinical findings of the trial. ^11^ Additionally, the more detailed data regarding healthcare utilisation and health-related quality of life for the hand OA patients included in this study is of potential use for future studies or models. Hence the detailed breakdown of the EQ-5D-5L responses and resource use at the different time points have been provided. The economic evaluation considered costs and outcomes encountered over a 12-month time horizon. Analysis over a longer period of time may identify further costs that occur in the long-term, such as drug monitoring costs. For instance, if HCQ is used in the long-term, patients require regular ophthalmology screening over time due to the associated risk of retinopathy. ^29^ Hence there are other potential costs that were not captured in the present analysis.

A low proportion of participants (31%) had complete economic data profiles. The cumulative nature of the costs and QALYs that constitute the complete case analysis means that the economic profile is considered incomplete if only one cost item is missing. The study included several resource use items, and the ‘other’ resource use responses were often left blank ( *i.e.* classed as missing), hence all data for the participant is lost using complete case analysis. The occurrence of missing data is, however, likely in economic evaluations that involve patient-level data, ^30^ and was dealt with in this economic evaluation using multiple imputation.

The analysis applied costs as accurately as possible to generate the overall costs for both study groups. However, assumptions were made where necessary, for example by applying medications categories in order to simplify the medication costing, rather than micro-costing at the individual medication level due to the high volume of medications reported. We acknowledge that for the secondary analysis there is the possibility of double counting with the over-the-counter medications and the separate medication costs included. A further point to note about the secondary analysis, which took a societal perspective, is that it did not cover an extensive list of items that could potentially feed into this perspective. This was due to keeping the questionnaire to a manageable length rather than including further questions which may have deterred participants from completing the questionnaire and reduced the questionnaire response rate. The key cost areas were therefore selected and included.

## Conclusion

This trial-based economic evaluation found that the use of HCQ for pain relief of patients with hand OA and moderate to severe pain was not a cost-effective management option when compared with placebo. Data from our study can be used to inform future studies in the area, regarding the use of healthcare services and the health-related quality of life of patients with hand OA.

## Data availability

### Underlying data

Full underlying (non-aggregated) data cannot be made publicly available since the ethics approval of this study does not cover openly publishing non-aggregated data.

In order to access this data, it must be requested from the corresponding author. Data requestors will have to provide: i) written description and legally binding confirmation that their data use is within the scope of the study; ii) detailed written description and legally binding confirmation of their actions to be taken to protect the data (e.g., with regard to transfer, storage, back-up, destruction, misuse, and use by other parties), as legally required and to current national and international standards (data protection concept); and iii) legally binding and written confirmation and description that their use of this data is in line with all applicable national and international laws (e.g., the General Data Protection Regulation of the EU).

## Reporting guidelines

Open Science Framework: CHEERS checklist for ‘Cost-effectiveness of hydroxychloroquine versus placebo for hand osteoarthritis: economic evaluation of the HERO trial’, https://doi.org/10.17605/OSF.IO/AQNWV. ^31^

Data are available under the terms of the Creative Commons Attribution 4.0 International License (CC-BY 4.0).

## Consent

All participants gave written informed consent prior to entering the trial.

## Ethical approval

Ethical approval was given by Leeds East Research Ethics Committee (reference number 12/YH/0151).
