# Supplementary material for: Cost-effectiveness of hydroxychloroquine versus placebo for hand osteoarthritis: economic evaluation of the HERO trial
Source: F1000Res. 2021 Aug 17;10:821. [Version 1] doi: 10.12688/f1000research.55296.1 (PMC8666991; doi:10.12688/f1000research.55296.1)
Supplement: Peer review history

# Peer review: Reviewer response for version 1

**DOI:** 10.5256/f1000research.58858.r100806

**Published:** 2021-12-10

**Authors:** David Epstein

**Affiliations:** 1 Department of Applied Economics, University of Granada, Granada, Spain

The authors conduct an economic evaluation alongside the RCT. There were no differences found between the groups in terms of hand pain or quality-of-life and no significant differences in costs.  Although there were no differences, it is nevertheless worthwhile publishing these results, in order to avoid "publication bias" and guide future research in this area. The study, in general, is well conducted and I have no comments on technical matters.  Rather than calculate an ICER, which implies some measurable difference in outcomes and costs, personally, I would interpret the results in the abstract and conclusions that there were no meaningful or statistically significant differences in any outcomes or costs at 1 year. 

 The authors do not discuss other therapies or research in this area and this contextual comparison would be useful.

Is the work clearly and accurately presented and does it cite the current literature?

Partly

If applicable, is the statistical analysis and its interpretation appropriate?

Yes

Are all the source data underlying the results available to ensure full reproducibility?

Partly

Is the study design appropriate and is the work technically sound?

Yes

Are the conclusions drawn adequately supported by the results?

Yes

Are sufficient details of methods and analysis provided to allow replication by others?

Yes

Reviewer Expertise:

Economic evaluation

I confirm that I have read this submission and believe that I have an appropriate level of expertise to confirm that it is of an acceptable scientific standard.

# Peer review: Reviewer response for version 1

**DOI:** 10.5256/f1000research.58858.r92218

**Published:** 2021-09-21

**Authors:** Elizabeth A. Stokes

**Affiliations:** 1 Health Economics Research Centre, Nuffield Department of Population Health, University of Oxford, Oxford, UK

This paper reports a within-trial cost-utility analysis (CUA) and cost-effectiveness analysis (CEA). The paper is clearly written, and appropriate methods have been used to conduct analyses. The text around interventions being cost-effective where findings are reported in terms of cost per QALY lost is very well explained.

 I have the following comments:

1. A CUA and a CEA were planned, did you pre-specify which was the primary analysis?
2. Introduction – first sentence – who is at-risk?
3. Resource use was captured at baseline, 6 and 12 months. Did the questionnaires at each of these time points ask participants to recall their resource use over the previous 6 months? Was resource use captured at baseline solely for the purpose of including baseline costs in the multiple imputation models?
4. Did you explore the missing at random assumption?
5. Costs – resource use was captured on day cases, but no unit cost for this is reported in Table 1. Were there no participants who reported a day case admission? Were hospital admissions not captured as there is no chance that this patient group would be admitted for hand OA? In the introduction, surgery is cited as one of the high costs in this patient group.
6. The mean difference between groups and 95% CI is presented in Table 3 for costs and Table 5 for EQ-5D utilities, but not in Table 2 for resource use? It would help the reader to include this.
7. Table 3 – did you consider separating medication costs into HCQ and other medications?
8. The time horizon for the CUA was 12 months but for the CEA was 6 months? While the primary clinical outcome of hand pain severity was measured at 6 months, this was also captured at 12 months. Why was your analysis for this outcome based on a shorter time horizon than the CUA analysis? Was a CEA over 12 months a pre-planned sensitivity analysis?

Is the work clearly and accurately presented and does it cite the current literature?

Yes

If applicable, is the statistical analysis and its interpretation appropriate?

Yes

Are all the source data underlying the results available to ensure full reproducibility?

Yes

Is the study design appropriate and is the work technically sound?

Yes

Are the conclusions drawn adequately supported by the results?

Yes

Are sufficient details of methods and analysis provided to allow replication by others?

Yes

Reviewer Expertise:

Health economics

I confirm that I have read this submission and believe that I have an appropriate level of expertise to confirm that it is of an acceptable scientific standard.
